# Investigation of Thermal Transport in Multi-Shaped Cu Nanomaterial-Based Nanofluids

**DOI:** 10.3390/ma13122737

**Published:** 2020-06-17

**Authors:** Adnan, Syed Zulfiqar Ali Zaidi, Umar Khan, Thabet Abdeljawad, Naveed Ahmed, Syed Tauseef Mohyud-Din, Ilyas Khan, Kottakkaran Sooppy Nisar

**Affiliations:** 1 Department of Mathematics, Mohi-ud-Din Islamic University, Nerian Sharif AJ&K 12080, Pakistan; adnan_abbasi89@yahoo.com; 2 Department of Mathematics, COMSATS University Islamabad, Abbottabad Campus, Abbottabad 22010, Pakistan; zzaidi@cuiatd.edu.pk; 3 Department of Mathematics and Statistics, Hazara University, Mansehra 21120, Pakistan; umar_jadoon@hu.edu.pk; 4 Department of Mathematics and General Sciences, Prince Sultan University, Riyadh 11586, Saudi Arabia; 5 Department of Medical Research, China Medical University, Taichung 40402, Taiwan; 6 Department of Computer Science and Information Engineering, Asia University, Taichung 40402, Taiwan; 7 Department of Mathematics, Faculty of Sciences, HITEC University Taxila Cantt, Taxila 47070, Pakistan; nidojan@gmail.com; 8 University of Multan, Multan 60000, Pakistan; syedtauseefs@hotmail.com; 9 Department of Mathematics, College of Science Al-Zulfi, Majmaah University, Al-Majmaah 11952, Saudi Arabia; 10 Department of Mathematics, College of Arts and Sciences, Prince Sattam bin Abdulaziz University, Wadi Aldawaser 11991, Saudi Arabia; n.sooppy@psau.edu.sa

**Keywords:** heat transfer, thermal conductivity, nanoparticles, shear stresses, RK scheme, HAM

## Abstract

The unsteady flow of H_2_O saturated by tiny nanosized particles with various shapes (platelets, blades, cylinders, and bricks) over a thin slit is reported. For this novel analysis, the influences of the magnetic field and heat generation/absorption are incorporated into the governing model. The dimensionless nanofluid model is attained after the successful implementation of similarity transformations. Then, Runge-Kutta and homotopy analysis algorithms are implemented for mathematical analysis, and the results are obtained by varying the main flow parameters. A decrease in nanofluid motion is observed for a stronger magnetic field (M). Additionally, nanofluid temperature β(η) increases for higher values of M. Decreasing trends in the shear stresses Re_x_^0.5^C_Fx_ are observed for the unsteadiness parameter S, and this declines with stronger M. Similarly, the local heat transfer rate Re_x_^−0.5^N_ux_ rises with the unsteady behavior of the fluid. It is observed that the nanofluid motion drops for variable thickness (λ) of the slit, whereas the motion becomes slower with stronger magnetic field effects (M).

## 1. Introduction

Heat transfer investigation has been a major concern for researchers, industrialists, scientists, and engineers. A remarkable amount of heat is necessary to accomplish many industrial processes, such as food and paper production. Therefore, the large amount of heat required to create these products has been a major problem for industrialists, engineers, scientists, and researchers. Unfortunately, carrier liquids are not able to produce the necessary amount of heat required to accomplish many production processes. Additionally, some researchers have proposed the replacement of conventional carrier liquids by a new sort of fluid that offers improved heat transfer properties. Thus, a new class of fluids has been introduced called “nanofluids” (Choi) [1].

The investigation of heat transport in nanofluids is a focus for engineers and industrialists. The beneficial heat transport properties of nanofluids have resolved problems faced by engineers. Nanofluids are compositions of regular liquids and tiny particles of different metals, oxides, carbon nanomaterials, ferromagnetic alloys, and alloys of various other metals. These particles are saturated in regular liquids in stable thermal equilibrium. Nanofluids have gained popularity for their extensive uses, including applications in medical sciences, chemistry, civil engineering, aerodynamics, manufacturing of aircraft, home appliances, electronics, and different computer chips.

Nanofluid models comprising various thermal conductance correlations are very difficult to tackle theoretically. The reason for this challenge is that thermal conductance correlations involve various properties, such as the diameter of the tiny particles, the effects of temperature, and molecular diameter. The resultant mathematical nanofluid models are highly nonlinear and coupled; therefore, such models are complex. However, mathematicians have proposed several mathematical techniques that help to tackle these models effectively.

The investigation of flow and thermal transport in nanofluids over a thin slit with variable thickness has versatile applications. Analysis of heat transfer and the impacts of Lorentz forces in flow regimes over a thin slit offers potential uses in the aforementioned industries. Nanofluid models that describe flow over a thin slit and comprise the effects of Lorentz forces are highly nonlinear and significant from an industrial point of view. Heat transfer in carrier fluids is very poor. Because of these poor heat transfer properties, carrier fluids have limited uses in industries.

Researchers have carried out heat transport investigations of nanofluids over a thin slit under various physical flow conditions. Recently, Shah et al. [2] described the influence of nonlinear radiative heat flux in a magnetized nanofluid thin film flow. They adopted analytical and numerical techniques for the solution and reported the results of nanofluid velocity and thermal transport. The second-law analysis and behavior of velocity and thermal fields resulting from an imposed magnetic field in a viscoelastic nanofluid are described in [3]. The researchers reported results for local heat transfer and discussed this comprehensively. A heat and mass transport analysis under the impact of variable Lorentz forces over a thin film was presented in [4]. They highlighted the behavior of mass and temperature for multiple values of these parameters. The influence of the Cattaneo–Christov constitutive model of a thin film flow of carbon nanotube-based nanofluids and entropy analysis were described in [5]. They treated the model by implementing a MATLAB built-in algorithm and published the results of the flow regimes. They pointed out that velocity and thermal profiles rise for high volume friction of carbon nanotubes. Furthermore, fruitful analyses of the flow and thermal behavior over a thin film were provided in [6,7].

Different theoretical models have been suggested in order to improve thermal transport in nanofluids. One of them is known as the Hamilton-Crosser model, which deals with particle shapes. The convective thermal transfer of nanofluids in the presence of variously shaped nanoparticles is described in [8]. These researchers also studied the impacts of varying nondimensional physical quantities on the flow behavior. Khan et al. [9] presented the flow of copper/water nanofluids in an oblique channel. The thermal behavior of a ferromagnetic fluid based on the convective nature of the flow conditions was reported in [10]. Sheikholeslami et al. [11] studied water-based nanofluids and evaluated the impacts of convective auxiliary conditions. They discussed this problem numerically and examined the effects of nondimensional quantities. The study of water saturated by carbon nanotubes was discussed in [12]. The impacts of the slip parameter and ohmic heating on Casson-flow properties over a convectively heated stretchable surface were discussed in [13,14], respectively. Incompressible flow was explored by considering viscous dissipation and thermal radiation in two nonparallel walls in [15,16]. The researchers performed analytical and numerical investigations for a nonlinear flow model and studied the flow field graphically. The flow of a magneto-nanofluid in a rotating channel is discussed in [17]. Recently, Athira et al. [18] explored the influence of silver nanosized particles on Jeffrey flow properties. Many other researchers have presented studies regarding nonlinear flow models for nanofluids (e.g., [19,20,21] and references therein).

A literature review indicates that thermal transport in colloidal fluids composed of various tiny particles (platelets, blades, bricks, and cylinders) over a thin slit has not been analyzed to date. Therefore, this study is presented to fill this significant research gap. Our aims are to examine the heat transfer behavior by incorporating the influence of Lorentz forces and heat generation/absorption in the energy constitutive relation. The model is formulated and reduced into the self-similar version by plugging in defined invertible transformations. The model is treated analytically and numerically over the domain of interest. The homotopy analysis method (HAM) and Runge–Kutta (RK) algorithm are merged with the shooting technique in the presented analysis. Then, the results are presented with the main parameters and discussed comprehensively. Finally, the key output of the study is highlighted in the conclusions section.

## 2. Materials and Methods

### 2.1. Model Formulation

#### 2.1.1. Statement and Geometry of the Model

**Figure 1:**
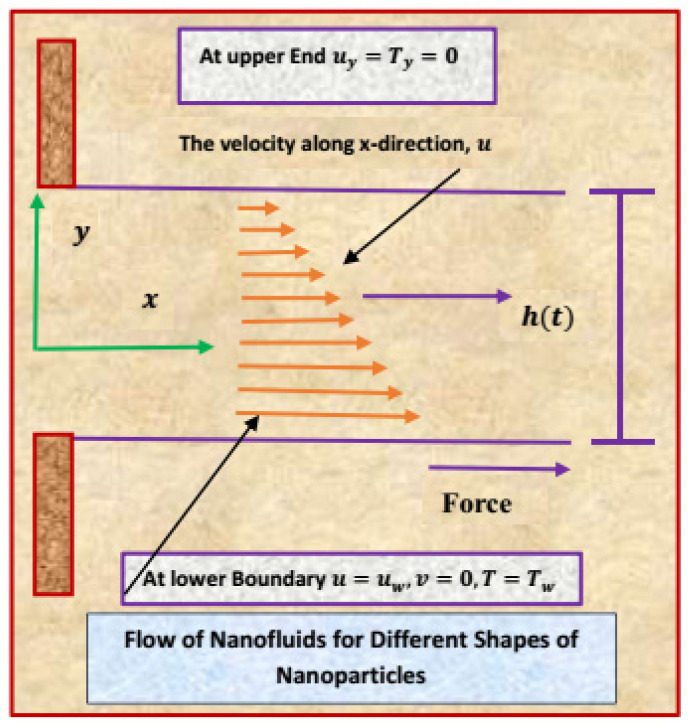
The flow of nanofluid composed of multiple nanomaterials.

In this model, 2D electrically conducting unsteady flow is considered. The sheet is positioned along the *x*-axis. The velocity in the horizontal direction is u_w_ = (1 − αt)^−1^ bx, where u_w_ depends on x and t. Furthermore, b and α are constant quantities. The temperature at the wall is T_s_(x, t) = (T_0_ − T_r_)/(1 − αt)^1.5^bx^2^(2ν_f_)^−1^. Here, reference and slit temperatures are denoted by T_0_ and T_r_, respectively. A time-dependent magnetic field is applied perpendicular to the slit with strength B_0_, where B(t) = B_0_/(√(1 − αt)). Moreover, the variable slit thickness is represented by h(t). Furthermore, it is assumed that the nanoparticles have the shapes of platelets, blades, cylinders, and bricks and that there is no slip condition between them. Figure 1 depicts the appropriate geometry of the particular nanofluid model.

#### 2.1.2. Governing Model and Similarity Transformations

The considered nanofluid model includes the following set of partial differential equations (PDEs) comprising the impact of Lorentz forces that describe the flow through a thin slit:

(1) $$\frac{\partial u}{\partial x}+\frac{\partial v}{\partial y}=0$$

(2) $$\rho_{\mathrm{nf}}\left(\frac{\partial u}{\partial t}+u\frac{\partial u}{\partial x}+v\frac{\partial u}{\partial y}\right)=\mu_{\mathrm{nf}}\;\left(\frac{\partial^{2}u}{\partial y^{2}}\right)-\sigma_{\mathrm{nf}}B^{2}\;u$$

(3) $${\left({\rho C}_{p}\right)}_{\mathrm{nf}}\left(\frac{\partial T}{\partial t}+u\frac{\partial T}{\partial x}+v\frac{\partial T}{\partial y}\right)=k_{\mathrm{nf}}\left(\frac{\partial^{2}T}{\partial y^{2}}\right)+q^{\prime \prime \prime }$$

Equation (1) describes mass conservation, and Equations (2) and (3) represent the well-known dimensional momentum and energy equations, respectively. Further, thermal conductivity is denoted by k_nf_, specific heat capacity is (ρC_p_)_nf_, effective electrical conductivity is σ_nf_, and the dynamic viscosity and density of the nanofluid are μ_nf_ and ρ_nf_, respectively. Furthermore, u and v represent the velocity in the horizontal and vertical positions, respectively. The time- and thermal-dependent sink/source quantity q‴ is given in the following formula:

(4) $$q^{\prime \prime \prime }={\left({x\nu }_{f}\right)}^{-1}k_{f}\left(T_{s}-T_{0}\right)u_{w}\left(x,t\right)\left(A_{1}\;F^{\prime }+B_{1}{\left(T_{s}-T_{0}\right)}^{-1}\left(T-T_{0}\right)\right)$$

Here, A_1_ and B_1_ represent the heat generation and absorption parameters, respectively. Furthermore, we used the following effective nanofluid models:

(5) $$\mu_{\mathrm{nf}}=\mu_{f}\;\left(1+a^{*}\phi +b\phi^{2}\right)$$

(6) $$k_{\mathrm{nf}}=k_{f}\left[\frac{k_{s}+\left(n-1\right)k_{f}+\left(n-1\right)\left(k_{s}-k_{f}\right)\phi }{k_{s}+\left(n-1\right)k_{f}-\left(k_{s}-k_{f}\right)\phi }\right]$$

(7) $$\frac{\rho_{\mathrm{nf}}}{\rho_{f}}=\left(1-\phi \right)+\frac{\phi \rho_{s}}{\rho_{f}}$$

(8) $$\frac{{\left({\rho C}_{p}\right)}_{\mathrm{nf}}}{{\left({\rho C}_{p}\right)}_{f}}=\left(1-\phi \right)+\frac{\phi {\left({\rho C}_{p}\right)}_{s}}{{\left({\rho C}_{p}\right)}_{f}}$$

Here, a* and b are constants, ϕ is the volumetric fraction, ρ_s_ shows the effective density of the nanoparticles, ρ_f_ is the density of the carrier fluid, and n = 3/ψ is the empirical shape factor. The thermal and physical properties are reported in [8]. The empirical shape factor, sphericity, and thermophysical attributes of the host liquid and Cu tiny particles are described in Table 1, Table 2 and Table 3, respectively.

The boundaries of the slit at y = 0 and y = h(t) are specified as follows:

(9) $$u{↓}_{y=0}=u_{w},\;v{↓}_{y=0}=0,\;T{↓}_{y=0}=T_{s}$$

(10) $$\frac{\partial u}{\partial y}{↓}_{y=h\left(t\right)}=0,\;v{↓}_{y=h\left(t\right)}=h_{t},\;\mathrm{and}\;\frac{\partial T}{\partial y}{↓}_{y=h\left(t\right)}=0$$

The suitable self-similar variables are defined in the following way:

(11) $$\eta =\frac{1}{\beta^{*}}\;{\left(\frac{b}{\nu_{f}\left(1-\alpha t\right)}\right)}^{\frac{1}{2}}y$$

(12) $$\varphi =\beta^{*}{\left(\frac{\nu_{f}b}{\left(1-\alpha t\right)}\right)}^{\frac{1}{2}}\mathrm{xF}\left(\eta \right)$$

(13) $$\beta \left(\eta \right)=\frac{T-T_{0}}{T_{s}-T_{0}}$$

(14) $$T_{s}=T_{0}-T_{r}\left({\mathrm{bx}}^{2}{\left(2\nu_{f}\right)}^{-1}\right){\left(1-\alpha t\right)}^{-1.5}\beta \left(\eta \right),\;\forall \;t<1/\alpha$$

(15) $$u=\frac{\partial \varphi }{\partial y},\;v=-\frac{\partial \varphi }{\partial x}$$

By using these similarity variables and models for nanofluids (given above), in the dimensional model for the nanofluids given by Equations (1)–(3), we get the following nondimensional flow model:

(16) $$F^{\prime \prime \prime }-\frac{\left(1-\phi \right)+\phi \left[\frac{\rho_{s}}{\rho_{f}}\right]}{\left(1+a^{*}\phi +b\phi^{2}\right)}\;\lambda \left(FF^{\prime \prime }-F^{\prime }{}^{2}-SF^{\prime }-s\frac{\eta }{2}F^{\prime \prime }\right)-M^{2}\frac{\left\{\frac{\left(\sigma_{s}+2\sigma_{f}\right)+2\left(\sigma_{s}-\sigma_{f}\right)\phi }{\left(\sigma_{s}+2\sigma_{f}\right)-\left(\sigma_{s}-\sigma_{f}\right)\phi }\right\}}{\left(1+a^{*}\phi +b\phi^{2}\right)}F^{\prime }=0$$

(17) $$\beta^{\prime \prime }-\mathrm{Pr}\frac{\left(1-\phi \right)+\phi \left[\frac{{\left({\rho C}_{p}\right)}_{s}}{{\left({\rho C}_{p}\right)}_{f}}\right]}{\left[\frac{k_{s}+k_{f}\left(n-1\right)+\left(n-1\right)\phi \left(k_{s}-k_{f}\right)}{k_{s}+\left(n-1\right)k_{f}-\left(k_{s}-k_{f}\right)\phi }\right]}\;\lambda \left(2F^{\prime }\beta -F\beta^{\prime }+\frac{S}{2}\left(3\beta +\eta \beta^{\prime }\right)\right)\;+\frac{1}{\left[\frac{k_{s}+\left(n-1\right)k_{f}+\left(n-1\right)\left(k_{s}-k_{f}\right)\phi }{k_{s}+k_{f}\left(n-1\right)-\phi \left(k_{s}-k_{f}\right)}\right]}\left(A_{1}F^{\prime }+B_{1}\beta \right)=0$$

The conditions at the boundaries of the slit are the following:

(18) $$F\left(\eta \right){↓}_{\eta =0}=0,\;F^{\prime }\left(\eta \right){↓}_{\eta =0}=1,\;\beta \left(\eta \right){↓}_{\eta =0}=1$$

(19) $$F^{\prime \prime }\left(\eta \right){↓}_{\eta =1}=0,\;\beta^{\prime }\left(\eta \right){↓}_{\eta =1}=0$$

The quantities incorporated in the model are the Prandtl number, Hartmann number, and unsteadiness parameter. Mathematical expressions for the aforementioned parameters are as follows:

$$\mathrm{Pr}=\frac{\mu_{f}{\left(c_{p}\right)}_{f}}{k_{f}},\;M^{2}=\frac{\sigma_{f}B_{0}^{2}\nu_{f}}{{b\mu }_{f}},\;S=\frac{\alpha }{b}.\;\mathrm{Also}\;\lambda =\beta^{2^{*}}$$

and β* is defined as

$$\beta^{*}=\frac{\mathrm{hb}{\left(\nu_{f}\right)}^{-1}}{{\left(1-\alpha t\right)}^{1/2}}$$

Physical quantities such as shear stresses and the local Nusselt number are of great interest from an engineering point of view. In their self-similar form, these quantities are as below:

(20) CFxRex=A2β*A1 F″(η)↓η=0

(21) Nux(Rex)−12=−A4β* β′(η)↓η=0

(22) $$A_{1}^{*}=\left(1-\phi \right)+\phi \left[\frac{\rho_{s}}{\rho_{f}}\right],\;A_{2}^{*}=1+a^{*}\phi +b\phi^{2},\;\mathrm{and}\;A_{4}^{*}=\left[\frac{k_{s}+\left(n-1\right)k_{f}+\left(n-1\right)\left(k_{s}-k_{f}\right)\phi }{k_{s}+\left(n-1\right)k_{f}-\left(k_{s}-k_{f}\right)\phi }\right]$$

where Rex=uwxνf is the local Reynolds number.

### 2.2. Mathematical Analysis

The particular model in this study is of a nonlinear nature. For this kind of model, closed solutions are difficult. The set of ordinary differential equations (ODEs) given by Equations (5) and (6) is highly nonlinear in nature and coupled. For this sort of system, exact solutions are infeasible. Thus, we tackled this problem by considering the flow of magneto-nanofluids analytically. For this purpose, we used the boundary value problem HAM (BVPH2.0). To initiate the package, the following estimates were made:

(23) $$F_{0}\left(\eta \right)=\eta$$

(24) $$\beta_{0}\left(\eta \right)=1$$

The supporting linear operators are ${ℒ}_{F}=\frac{d^{3}F}{{d\eta }^{3}}$ and ${ℒ}_{\beta }=\frac{d^{2}\beta }{{d\eta }^{2}}$, respectively. These operators obey the linear property:

(25) $${ℒ}_{F}\left(N_{1}^{*}+N_{2}^{*}\left(\eta \right)+N_{3}^{*}{\left(\eta \right)}^{2}\right)=0$$

(26) $${ℒ}_{\beta }\left(N_{4}^{*}+N_{5}^{*}\left(\eta \right)\right)=0$$

where $N_{k}^{*}$ (k=1…5) is a constant.

The auxiliary parameters for the velocity and temperature ($\hbar_{F}$ and $\hbar_{\beta }$) embedded in the solution play vital roles in the convergence. The following mathematical formulae are used to calculate these parameters:

(27) $$Y_{m^{*}}{,}_{1}\left(\hbar_{f}\right)=\frac{1}{B^{*}}\overset{B^{*}}{\underset{n=0}{\sum }}{\left\{N_{F}\left(\overset{m^{*}}{\underset{j=0}{\sum }}F_{j^{*}}\left(n∆x^{*}\right)\right)\right\}}^{2}$$

(28) $$Y_{m^{*}}{,}_{2}\left(\hbar_{\beta }\right)=\frac{1}{B^{*}}\overset{B^{*}}{\underset{n=0}{\sum }}{\left\{N_{\beta }\left(\overset{m^{*}}{\underset{j=0}{\sum }}\beta_{j^{*}}\left(n∆x^{*}\right)\right)\right\}}^{2}$$

where $∆x^{*}=\frac{1}{B^{*}}$. The solutions of the model are calculated over the domain of interest, and the values of auxiliary parameters are also determined for varying relevant parameters to validate the applied method. Table 4 and Table 5 present the optimal values and solution of the model. Further, both analytical and numerical solutions show excellent agreement.

## 3. Physical Interpretation of the Results

Changes in the main flow quantities, such as the Prandtl number (Pr), imposed magnetic field (M), and unsteadiness parameter (S), are significant in the behavior of temperature, velocity, and the local heat transfer rate for the model under consideration.

**Figure 2:**
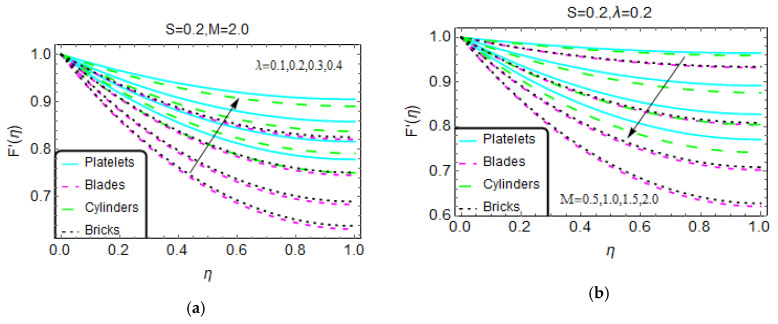
The velocity pattern for different values of (**a**) λ and (**b**) M.

The changes in the velocity F’(η) for λ and the magnetic number M are shown in Figure 2 over the region of interest. It is noted that the velocity of the nanofluid for various geometries over the thin slit rises for higher λ. At η = 0, these are almost negligible for multiple nanofluids based on the tiny particles’ geometries. However, the velocity gradually rises towards the region η = 1.

**Figure 3:**
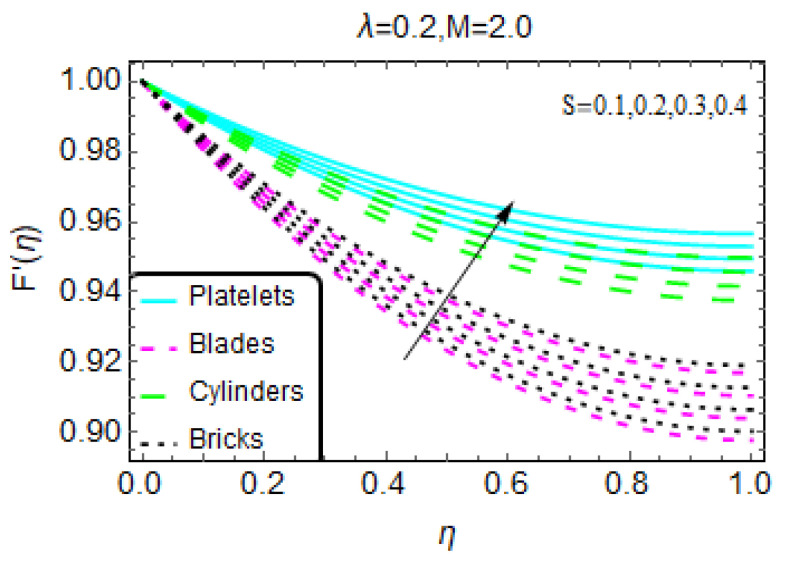
The velocity pattern for different values of S.

The Lorentz force is of great importance for its uses in multiple industrial production processes and in various engineering disciplines. The influence of the magnetic number due to the imposed magnetic field on the nanofluid velocity F’(η) is illustrated in Figure 2b. Decreasing values of F’(η) are observed. Physically, this means that the applied magnetic field opposes the nanofluid motion. Consequently, the momentum drops, which leads to a drop in the velocity F’(η). Near the higher end of the range (η = 1), an abrupt decrement in F’(η) is detected because, with these values, the magnetic field is very strong in comparison with lower values (η = 0). Further, for the tiny particles with a blade geometry, values of F’(η) rapidly decrease. Figure 3 shows the influences of the unsteadiness number S on F’(η) for multiple values. These results show that a more unsteady nanofluid tends to be associated with increasing velocity F’(η), and an abrupt increase in F’(η) is observed for tiny particles with a platelet geometry. The velocity F’(η) increases very slowly for a nanofluid with blade-type tiny particles.

**Figure 4:**
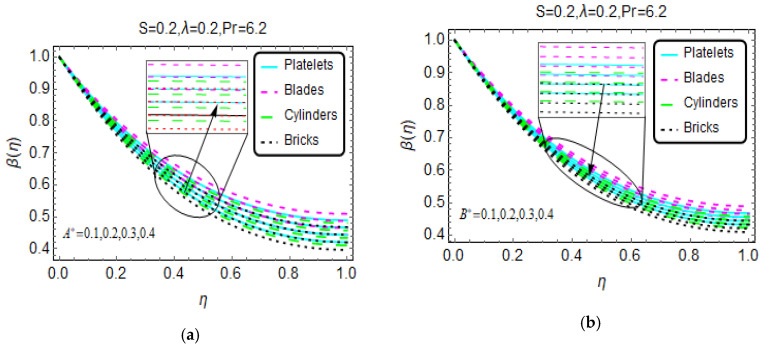
The temperature pattern for different values of (**a**) $A^{*}$ and (**b**) $B^{*}$.

**Figure 5:**
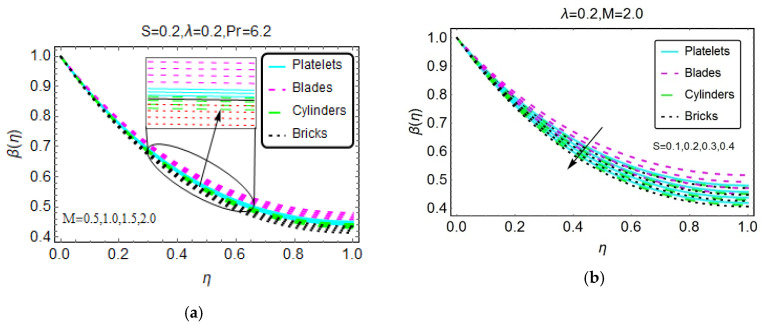
The temperature pattern for different values of (**a**) M and (**b**) S.

**Figure 6:**
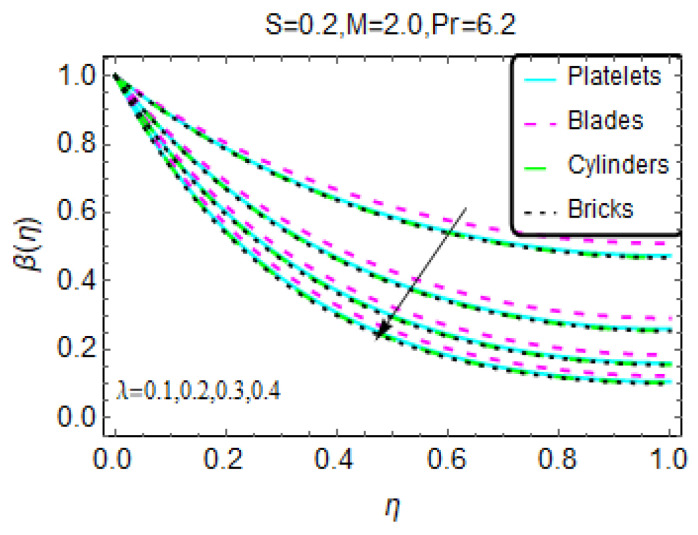
The temperature pattern for different values of λ.

Nanofluids are very popular for their effective heat transfer properties. Figure 4, Figure 5 and Figure 6 present the influences of the considered flow parameter on the temperature of various nanofluids based on tiny particles with different geometries. The Prandtl number is fixed at 6.2 because water is taken as the host liquid.

Figure 4 depicts the changes in temperature β(η) for the heat source/sink parameter. The results show that as a result of the internal heat source, the temperature increases for nanofluids composed of various tiny particles. Physically, this is due to the internal heat source, which provides extra energy to the nanofluid molecules, and this additional energy leads to the increase in temperature β(η). On the other side, B* restricts the nanofluid temperature β(η) abruptly near the region η = 1. A higher magnetic number appears to be beneficial for thermal transport, and a greater increase in temperature is observed for the nanofluid composed of blade-shaped tiny particles. Near η = 0, these influences are almost inconsequential because the effects of the imposed Lorentz forces are weaker for these values. Moreover, for a more unsteady nanofluid, the temperature β(η) drops. The behavior of β(η) for higher λ is shown in Figure 6. The stronger λ decreases the nanofluid temperature β(η).

**Figure 7:**
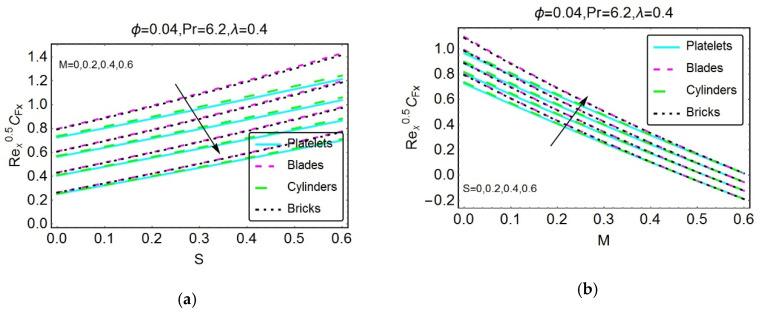
The variations in the skin friction coefficient for different values of (**a**) M and (**b**) S.

**Figure 8:**
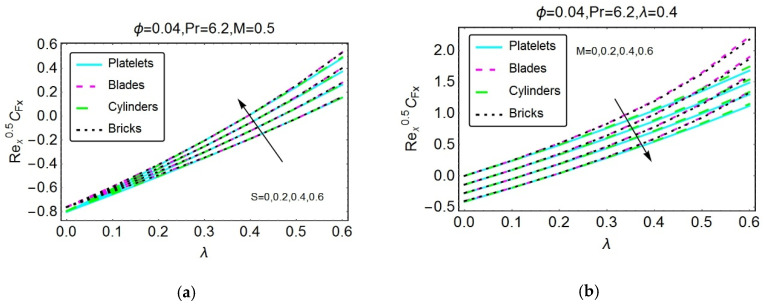
The variations in the skin friction coefficient in the y-direction for different values of (**a**) S and (**b**) M.

**Figure 9:**
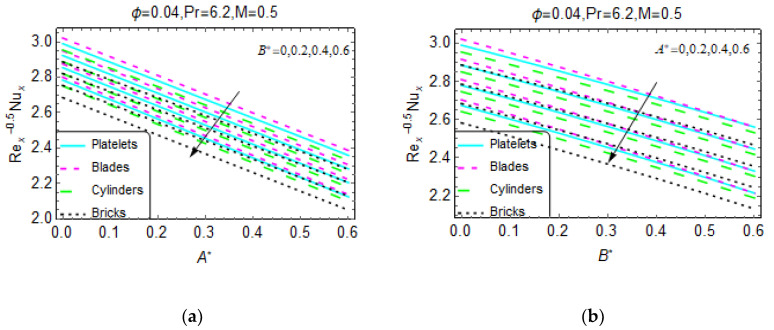
The variations in the Nusselt number for different values of (**a**) $B_{1}$ and (**b**) $A_{1}$.

**Figure 10:**
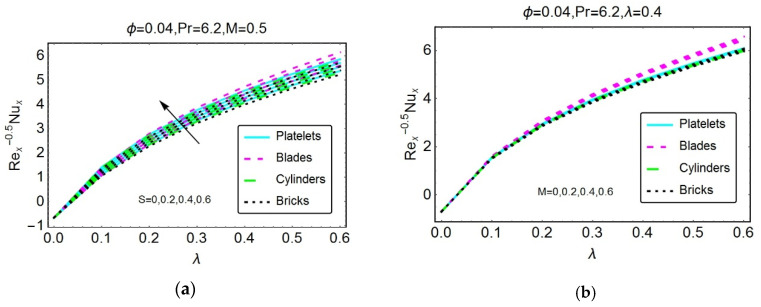
Variations in the Nusselt number for different values of (**a**) S and (**b**) M.

**Figure 11:**
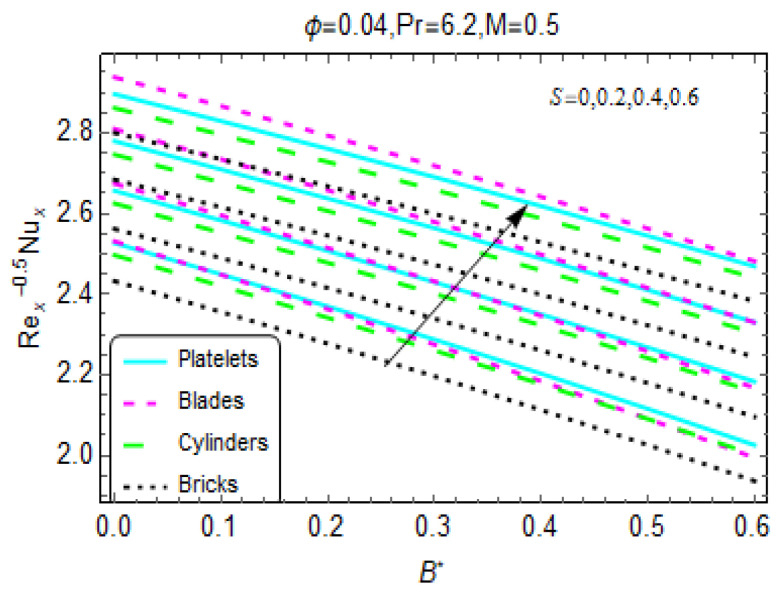
Variations in the Nusselt number for different values of S.

Studying wall shear stresses and local heat transportation is significant from industrial and engineering points of view. Therefore, Figure 7, Figure 8, Figure 9, Figure 10 and Figure 11 are presented to analyze the behavior of shear stresses and heat transportation for multiple flow quantities. The stronger magnetic field restricts the wall shear stresses because the high impact of the magnetic field causes the motion of the nanofluids to decline, and consequently, the transport of shear stresses drops. Rapid decreases are detected for nanofluids composed of cylinder-shaped tiny particles. For more unsteady flow, maximum shear stresses at the wall are detected because of the increased unsteadiness of the nanofluid. These findings are illustrated in Figure 7a,b, respectively. The shear stresses S versus λ and M versus λ are plotted in Figure 8a,b, respectively.

Figure 9a,b express the local heat transportation rate at the wall. It is noted that Re_x_^−0.5^N_ux_ declines for B* and A*. Rapid decreases are observed for the nanofluid composed of brick-shaped tiny particles in both cases. Further, nanofluids that are more unsteady favor heat transportation at the wall. These effects are shown in Figure 10 and Figure 11.

## 4. Validation of the Analysis

The results of comparative analysis for some of the involved parameters are presented in Table 6. It is noteworthy to mention that for zero volumetric friction of the nanoparticles, the present model is reduced to the conventional flow model. Therefore, we compared the results with those of conventional models and observed that the presented results are reliable.

## 5. Conclusions

A thermal transport analysis in nanofluids for multiple shapes of tiny particles in the presence of Lorentz forces and heat generation/absorption is reported. The flow is carried out over an unsteady thin slit. The effects of the main flow parameters on the velocity and temperature behavior are illustrated. It is observed that the nanofluid velocity F’(η) rises, and stronger effects of the imposed magnetic field resist the motion of the fluid. The velocity of nanofluids composed of the platelet- and blade-shaped tiny particles is highest among the studied particle shapes. The nanofluid temperature β(η) increases for a more magnetized fluid, and the unsteadiness parameter S decreases it. Further, an increase in the shear stresses is observed for higher values of M, whereas they decrease with the unsteadiness parameter. On the other hand, the effects of S for the local heat transfer rate are strong. Moreover, it is observed that the nanofluid comprising blade-shaped nanomaterial has a high heat transport capacity and is thus promising for industrial uses.

## 6. Achievements

A comparative heat transfer analysis in the nanofluids comprising the tiny particles with various shapes (blades, cylinders, bricks, and platelets) is reported. From the presented results, it is observed that the nanofluid comprising the blade-shaped nanomaterial has excellent heat transport properties. Therefore, these materials are better for practical applications to overcome the heat transport issues of engineers and industrialists.

## Figures and Tables

**Table 1:** Empirical shape factors.

Model	Platelets	Blades	Cylinders	Bricks
$a^{*}$	37.1	14.6	13.5	1.9
b	612.6	123.3	904.4	471.4

**Table 2:** Sphericity (ψ) or the nanoparticles.

Model	Platelets	Blades	Cylinders	Bricks
ψ	0.52	0.36	0.62	0.81

**Table 3:** Thermophysical properties.

Model	ρ (kgm^−3^)	$c_{p}$ (kg^−1^k^−1^)	k (wm^−1^k^−1^)	$\beta^{*}$ (10^−5^k^−1^)
$H_{2}O$	997.1	4179	0.613	21
Cu	8933	385	401	1.67

**Table 4:** The values of auxiliary parameters.

λ	S	M	$A_{1}$	$B_{1}$	$\hbar_{f}$	$\hbar_{\beta }$	Error
0.2	0.2	0.2	0.2	0.2	−0.65145	−0.72236	$1.24527\times 10^{-10}$
0.4					−0.86694	−0.56591	$7.61662\times 10^{-7}$
0.6					−1.69158	−0.53692	$-3.60051\times 10^{-4}$
0.2	0.4				−0.70535	−0.68526	$-4.20735\times 10^{-10}$
	0.6				−1.52688	−0.79938	$-3.14081\times 10^{-7}$
	0.2	0.4			−0.97360	−0.72343	$-9.49486\times 10^{-10}$
		0.6			−1.21863	−0.69243	$-6.768686\times 10^{-8}$
		0.2	0.4		−1.02047	−0.73573	$-3.14275\times 10^{-10}$
			0.6		−0.66141	−0.71472	$1.77114\times 10^{-10}$
			0.2	0.4	−1.57109	−0.96331	$-1.25138\times 10^{-7}$
				0.6	−1.68925	−1.06871	$-8.10655\times 10^{-7}$

**Table 5:** Solutions of the model.

η ↓	F(η)			β(η)		
	HAM	Numerical	Error	HAM	Numerical	Error
0.0	0.0000	0.0000	0.0000	1.0000	1.0000	0.0000
0.1	0.10013	0.10013	$9.722400\times 10^{-10}$	0.87309	0.87309	$5.333840\times 10^{-8}$
0.2	0.20050	0.20050	$1.761650\times 10^{-9}$	0.76556	0.76556	$4.908750\times 10^{-8}$
0.3	0.30109	0.30109	$3.020970\times 10^{-9}$	0.67574	0.67574	$6.497240\times 10^{-8}$
0.4	0.40187	0.40187	$4.770370\times 10^{-9}$	0.60206	0.60206	$3.247030\times 10^{-8}$
0.5	0.50282	0.50282	$6.911800\times 10^{-9}$	0.54305	0.54305	$2.215420\times 10^{-8}$
0.6	0.60390	0.60390	$9.409160\times 10^{-9}$	0.49733	0.49733	$1.406580\times 10^{-8}$
0.7	0.70509	0.70509	$1.220780\times 10^{-8}$	0.46363	0.46363	$7.046780\times 10^{-9}$
0.8	0.80637	0.80637	$1.524420\times 10^{-8}$	0.44079	0.44079	$1.502500\times 10^{-9}$
0.9	0.90770	0.90770	$1.844790\times 10^{-8}$	0.42776	0.42776	$6.340190\times 10^{-9}$
1.0	1.00906	1.00906	$2.174040\times 10^{-8}$	0.42361	0.42361	$3.568580\times 10^{-9}$

**Table 6:** Comparison with scientific literature for $\phi =0,\;M=0,\;a^{*}=0,\;b=0$.

S	Presented Results		[22]		Present Results		[23]	
	λ	F″(0)	λ	F″(0)	λ	F″(0)	λ	F″(0)
1.4	0.674089	−1.01278	0.674089	−1.01278	0.674097	−1.01278	0.674097	−1.01278
1.6	0.331976	−0.642398	0.331976	−0.64240	0.331977	−0.642399	0.331977	−0.64241
1.8	0.127013	−0.309137	0.127013	−0.309138	0.127014	−0.309139	0.127014	−0.339138

## Nomenclature

u, v	Velocities in the x- and y-directions, respectively (m/s)
x, y	Coordinates
T_o_ and T_r_	Temperature at the slit surface and reference temperature, respectively (K)
B_o_	Magnetic field (T)
α and b	Constants
nf	Denotes the nanofluid
$\rho_{\mathrm{nf}}$	Nanofluid density (kg/m^3^)
$\rho_{s}$ and $\rho_{f}$	Densities of the tiny particles and host liquid, respectively (kg/m^3^)
$\mu_{\mathrm{nf}}$	Dynamic viscosity of the nanofluid (kg/ms)
$\sigma_{\mathrm{nf}}$	Electrical conductivity of the nanofluid (S/m)
$\sigma_{s}$ and $\sigma_{f}$	Electrical conductivities of the tiny particles and host liquid, respectively (S/m)
${\left({\rho C}_{p}\right)}_{\mathrm{nf}}$	Specific heat capacity of the nanofluid (J/kg K)
${\left(C_{p}\right)}_{f}$, ${\left(C_{p}\right)}_{s}$	Specific heat capacities of the liquid and tiny particles, respectively (J/kg K)
$k_{\mathrm{nf}}$	Thermal conductivity of the nanofluid (W/m K)
$k_{f}$ and $k_{s}$	Thermal conductivities of the liquid and tiny particles, respectively (W/m K)
n	Shape factor of the particles
ϕ	Volume fraction of the particles
η	Invertible variable
F(η)	Dimensionless velocity
β(η)	Dimensionless temperature
λ	Dimensionless slit thickness
φ	Stream function
Pr	Prandtl number
M	Magnetic number
S	Unsteadiness number
C_Fx_	Dimensionless skin friction coefficient
Nu_x_	Dimensionless Nusselt number
${ℒ}_{F}$ and ${ℒ}_{\beta }$	Linear operators
